# Glucose Evokes Rapid Ca^2+^ and Cyclic AMP Signals by Activating the Cell-Surface Glucose-Sensing Receptor in Pancreatic β-Cells

**DOI:** 10.1371/journal.pone.0144053

**Published:** 2015-12-02

**Authors:** Yuko Nakagawa, Masahiro Nagasawa, Johan Medina, Itaru Kojima

**Affiliations:** Department of Cell Biology, Institute for Molecular & Cellular Regulation, Gunma University, Maebashi, Japan; Tohoku University, JAPAN

## Abstract

Glucose is a primary stimulator of insulin secretion in pancreatic β-cells. High concentration of glucose has been thought to exert its action solely through its metabolism. In this regard, we have recently reported that glucose also activates a cell-surface glucose-sensing receptor and facilitates its own metabolism. In the present study, we investigated whether glucose activates the glucose-sensing receptor and elicits receptor-mediated rapid actions. In MIN6 cells and isolated mouse β-cells, glucose induced triphasic changes in cytoplasmic Ca^2+^ concentration ([Ca^2+^]_c_); glucose evoked an immediate elevation of [Ca^2+^]_c_, which was followed by a decrease in [Ca^2+^]_c_, and after a certain lag period it induced large oscillatory elevations of [Ca^2+^]_c_. Initial rapid peak and subsequent reduction of [Ca^2+^]_c_ were independent of glucose metabolism and reproduced by a nonmetabolizable glucose analogue. These signals were also blocked by an inhibitor of T1R3, a subunit of the glucose-sensing receptor, and by deletion of the T1R3 gene. Besides Ca^2+^, glucose also induced an immediate and sustained elevation of intracellular cAMP ([cAMP]_c_). The elevation of [cAMP]_c_ was blocked by transduction of the dominant-negative G_s_, and deletion of the T1R3 gene. These results indicate that glucose induces rapid changes in [Ca^2+^]_c_ and [cAMP]_c_ by activating the cell-surface glucose-sensing receptor. Hence, glucose generates rapid intracellular signals by activating the cell-surface receptor.

## Introduction

Secretion of insulin is regulated by nutrients, neurotransmitters and hormones in pancreatic β-cells [1]. Among them, glucose is a primary stimulator of insulin secretion and is able to induce secretion by itself. Thus, when ambient glucose concentration rises, insulin secretion is initiated after a certain lag period [1]. The mechanism by which glucose stimulates insulin secretion has been investigated extensively for several decades [1, 2]. It was shown some decades ago that glucose induces complex changes in ion fluxes and membrane potential [3–6]. The resting membrane potential of mouse β-cells is between -60 and -70 mM [3–5], which is determined mainly by high permeability of K^+^. Elevation of ambient glucose leads to a gradual depolarization of 10 to 15 mV, which is followed by an initiation of action potentials. Initial depolarization induced by glucose is brought about by a decrease in K^+^ permeability of the plasma membrane. It is now known that glucose enters the cells, is metabolized through the glycolytic pathway and in mitochondria, and the resultant increase in ATP/ADP ratio causes closure of the ATP-sensitive K^+^ channel (K_ATP_ channel) [2, 5–7]. Closure of the K_ATP_ channel leads to gradual depolarization to a threshold, at which action potential driven by Ca^2+^ is initiated [4, 5, 7, 8]. Since it takes a minute or more for glucose to be metabolized, action potential starts after one to several minutes of lag time [7–8]. After the initial burst of action potential, the membrane potential returns to the level slightly below the resting potential, which is followed by cyclic changes in the membrane potential [4–6].

When changes in cytoplasmic Ca^2+^ concentration ([Ca^2+^]_c_) are monitored in pancreatic β-cells, the addition of a high concentration of glucose reduces [Ca^2+^]_c_ rather rapidly [9–11]. This initial decrease in [Ca^2+^]_c_ lasts for a few minutes and is followed by an oscillatory elevation of [Ca^2+^]_c_ [9–11]. The initial decrease in [Ca^2+^]_c_ is thought to be due to sequestration of Ca^2+^ mainly to endoplasmic reticulum (ER) via the ER Ca^2+^ pump (SERCA) [12, 13]. In fact, initial decrease in [Ca^2+^]_c_ is accompanied by an increase in Ca^2+^ concentration in ER [14, 15]. The role of this sequestration of Ca^2+^ to ER is not totally certain but it may be important for subsequent loading of Ca^2+^ into mitochondria. More importantly, the exact mechanism by which glucose stimulates sequestration of calcium into ER is not certain at present.

Besides changes in Ca^2+^, glucose also increases cyclic 3’, 5’ AMP (cAMP) in pancreatic β-cells [16–18]. Elevation of cytoplasmic cAMP concentration ([cAMP]_c_) induced by a high concentration of glucose has been thought to be secondary to elevation of [Ca^2+^]_c_ [18, 19]. In fact, pancreatic β-cells express adenylate cyclase (AC) isoforms, ACIII and ACVIII [20, 21]. ACVIII is a Ca^2+^-calmodulin-activated AC and is also regulated by G_s_. Presumably, elevation of [Ca^2+^]_c_ activates calcium-dependent AC such as ACVIII, and increases production of cyclic AMP [19]. However, in a study using islets obtained from transgenic mice expressing a cAMP sensor Epac1-camps, Kim et al. [22] showed that glucose evoked a rapid elevation of [cAMP]_c_, which preceded elevation of [Ca^2+^]_c_. This observation raises a possibility that increase in [cAMP]_c_ is rapid and at least partly independent of elevation of [Ca^2+^]_c_.

We have shown recently that subunits of the sweet taste receptor [23] are expressed in pancreatic β-cells [24]. Specifically, T1R3 subunit is abundantly expressed in β-cells while the protein expression of T1R2 is negligible [25]. Furthermore, the actions of sweet molecules are blocked by knockdown of T1R3 whereas knockdown of T1R2 was without effect. Based on these observations, we have speculated that a homodimer of the T1R3 functions as a cell-surface glucose-sensing receptor. Alternately, a heterodimer of T1R3 and another class C G protein-coupled receptor (GPCR) may function as a glucose-sensing receptor [26]. This receptor is activated by glucose and a nonmetabolizable analogue 3-*O*-methylglucose [26, 27]. Interestingly, activation of the glucose-sensing receptor by glucose facilitates its own metabolism in pancreatic β-cells [27]. Since activation of this receptor by artificial sweeteners leads to elevations of [Ca^2+^]_c_ and [cAMP]_c_ in β-cells [24], it is reasonable to speculate that glucose modulates [Ca^2+^]_c_ and [cAMP]_c_ in β-cells by acting on the glucose-sensing receptor. The present study was conducted to investigate whether or not the glucose-sensing receptor is involved in glucose-evoked intracellular signals. If this is the case, immediate changes in [Ca^2+^]_c_ and [cAMP]_c_ should be observed. We therefore investigated whether glucose elicits immediate actions on [Ca^2+^]_c_ and/or [cAMP]_c_ in pancreatic β-cells. The results show that glucose induces immediate changes in [Ca^2+^]_c_ and [cAMP]_c_ by acting on the glucose-sensing receptor.

## Materials and Methods

### Chemicals

Nifedipine, N-methyl-D-glucamine (NMDG) and 3-*O*-methylglucose were purchased from Wako Pure Chemical Industries, Ltd (Tokyo, Japan).

### Cell Culture

MIN6 cells (passages 16–21) [28] were grown in Dulbecco’s modified Eagle’s medium ‘high glucose’ (Wako Pure Chemical Industries), 50 μM β-mercaptoethanol, 1X Penicillin-Streptomycin Solution (Wako Pure Chemical Industries, Ltd) and 15% fetal bovine serum (Sigma-Aldrich, St. Louis, MO) and maintained in a humidified incubator of 95% air and 5% CO_2_ at 37°C.

### Animals

The animal experiment was approved by the Animal Experiment and Ethics Committee, Gunma University School of Medicine (#25–0112), and was conducted according to the guidelines for animal care issued by the Committee. B6; 129-Tas1r3 <tm1Csz>/J mice were purchased from the Jackson Laboratory (Bar Harbor, ME). They were kept in an experimental animal facility controlled at 23°C room temperature with a 12-hr light and dark cycle, and with free access to standard chow and water. The animal experiment was conducted according to the guidelines for animal care issued by Animal Experiment and Ethics Committee, Gunma University School of Medicine.

### Preparation of Pancreatic Islets

Islets were isolated from mouse pancreases using collagenase (Sigma-Aldrich). Single islet cells were prepared by shaking the islets in a Ca^2+^ free HKR buffer (129 mM NaCl, 5 mM NaHCO_3_, 4.7 mM KCl, 1.2 mM KH_2_PO_4_, 1.2 mM MgSO_4_, 0.1% BSA and 10 mM HEPES/ NaOH [pH 7.4]) [29]. The single cells were plated on a 35 mm glass bottom culture dish (Mat Tek, Ashland, MA) coated with collagen (Cellmatrix Type I-C, Nitta Gelatin Inc., Osaka, Japan) and cultivated in Dulbecco’s modified Eagle’s medium ‘low glucose’ (Wako Pure Chemical Industries) containing 1X Penicillin-Streptomycin Solution and 10% FBS.

### Solution

Hanks’ balanced salt solution (HBSS) contained 1.3 mM CaCl_2_, 5.4 mM KCl, 0.44 mM KH_2_PO_4_, 0.5 mM MgCl_2_, 0.38 mM MgSO_4_, 138 mM NaCl, 0.34 mM Na_2_HPO_4_, 2.8 mM D-glucose and 20 mM HEPES/ NaOH (pH 7.4). The Ca^2+^-free extracellular solution was prepared by removing CaCl_2_ and by adding 0.2 mM EGTA. To remove extracellular sodium, sodium was replaced by NMDG.

### Measurement of cAMP and Activation of PKC

MIN6 cells were transiently transfected with plasmid encoding the cAMP indicator Epac1-camps [30] kindly provided by Dr. Lohse of the University of Würzburg (Germany) or the myristoylated alanine-rich C kinase substrate (MARCKS) fused with a green fluorescent protein (GFP), MARCKS-GFP [31]. The transfected cells were used to measured cAMP levels and PKC activation as previously described [24]. Knockdown of T1R3 was performed as described previously [29].

### Imaging of Cytoplasmic Ca^2+^

Measurement of [Ca^2+^]_c_ was performed by using a fluorescent Ca^2+^ indicator Fluo-8 (AAT Bioquest, Sunnyvale, CA). MIN6 cells and isolated β-cells were loaded with 2 μM Fluo-8/ AM dissolved in HBSS for 20 min at room temperature. To monitor [Ca^2+^]_c_ using Cameleon-nano15, MIN6 cells were transfected with PM-Cameleon-nano15 [32] by electroporation as described previously [27]. The cells were placed on a 35 mm glass bottom culture dish. The cells were visualized with an Olympus UPlanAPO 10x Water Objective lens (Olympus, Tokyo, Japan). To detect fluorescence images, we used AQUACOSMOS/ASHURA, 3CCD based fluorescence energy transfer imaging system (Hamamatsu Photonics, Hamamatsu, Japan). Fluo-8 fluorescence was obtained by a U-MGFPHQ cube (Olympus), and expressed as the ratio of cytosolic fluorescence and initial intensity (F/F_0_). PM-Cameleon-nano15 fluorescence was obtained by a 440AF21 excitation filter (Omega Optical, Brattleboro, VT) and DM455DRLP dichroic mirror (Omega optical) and expressed as the ratio of CFP/YFP. These Images were captured with at a C7780-22 ORCA3CCD camera (Hamamatsu Photonics) at 10-second intervals.

### Measurement of DAG

To assess diacylglycerol (DAG) production, MIN6 cells were transfected with C1-tagged monomeric red fluorescent protein (C1_2_-mRFP) [33] by electroporation as described previously [27]. DAG levels were measured by using a prism-based TIRFM analysis [33].

### Statistical Analysis

Values are expressed as mean ± SE. Statistical analysis was done by using Mann-Whitney’s U-test. A *p* value of less than 0.05 was considered statistically significant.

## Results

### Effect of Glucose on Subplasmalemmal Free Calcium Concentration

**Fig 1:**
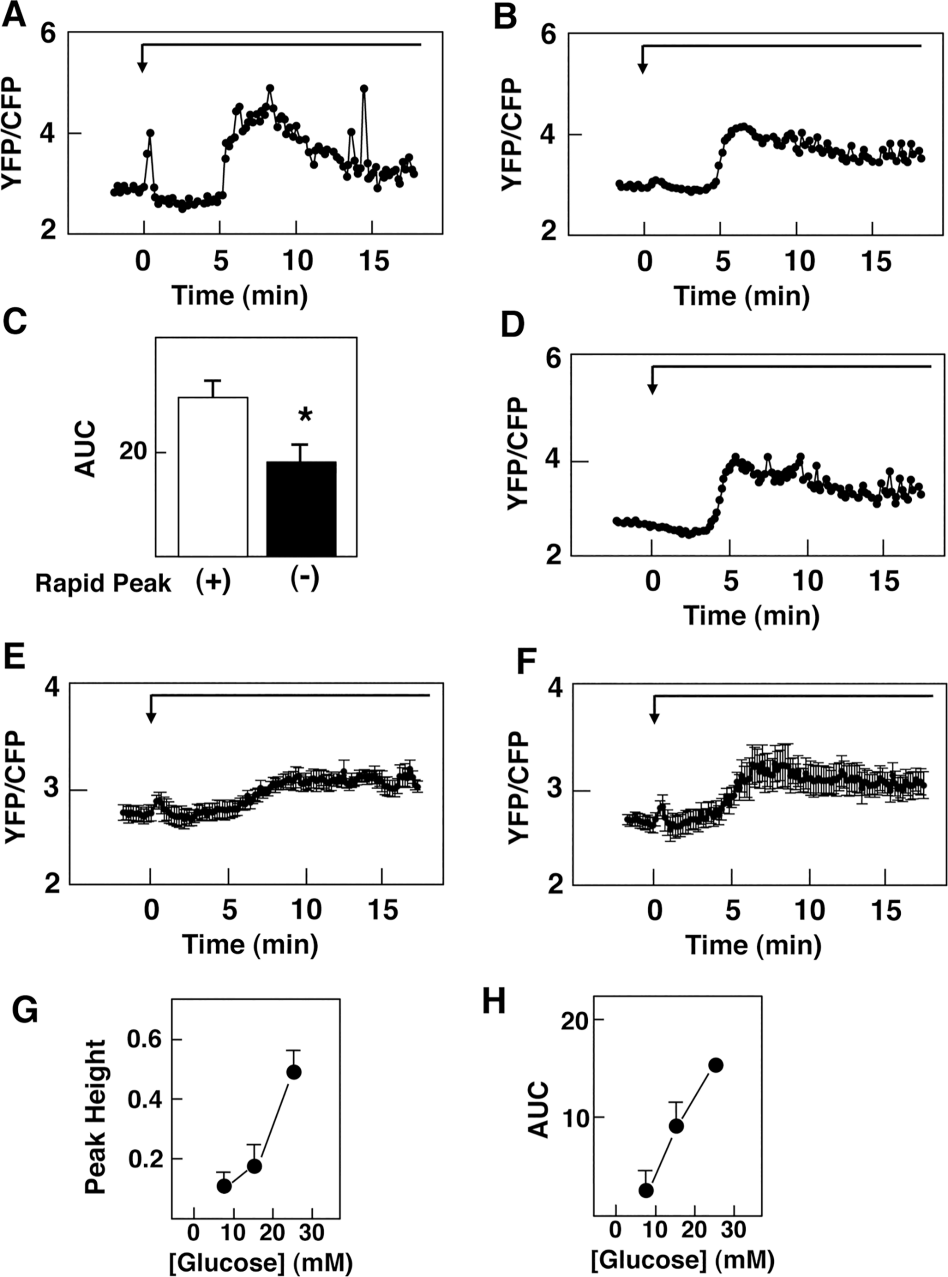
Effect of Glucose on [Ca^2+^]_c_ in MIN6 Cells. A: M-Cameleon-nano15-expressing cells were incubated in HBSS containing 2.8 mM glucose. The cells were then stimulated by 25 mM glucose as indicated by the arrow and changes in [Ca^**2+**^]_c_ were monitored. The result is a representative of those obtained in more than 300 cells from 10 experiments. B: Cells were treated as indicated above and a typical trace without the initial rapid peak is presented. The result is a representative of those obtained in more than 200 cells from 10 experiments. C: AUC of the oscillatory elevation of [Ca^**2+**^]_c_ (between 5 and 15 min) was calculated in A and B. Values are the mean ± SE of ten experiments. *: P< 0.01 vs ‘with rapid peak’. D: PM-Cameleon-nano15-expressing cells were also loaded with fura-2 and stimulated by 25 mM glucose as indicated by the arrow. Changes in [Ca^**2+**^]_c_ were monitored by using PM-Cameleon-nano15. E, F: PM-Cameleon-nano15-expressing cells were incubated in HBSS containing 2.8 mM glucose. Then cells were stimulated by 8.3 mM (E) or 16.7 mM (F) glucose and changes in [Ca^**2+**^]_c_ were monitored. Results obtained in cells presenting a rapid peak of [Ca^**2+**^]_c_ were shown. The results are the mean ± SE for 10 determinations. G, H: PM-Cameleon-nano15-expressing cells were stimulated by various concentrations of glucose as shown in A, E and F and the peak height of the rapid peak (G) and AUC from 5 to 10 min were measured. Values are the mean ± SE for 10 determinations.

**Fig 2:**
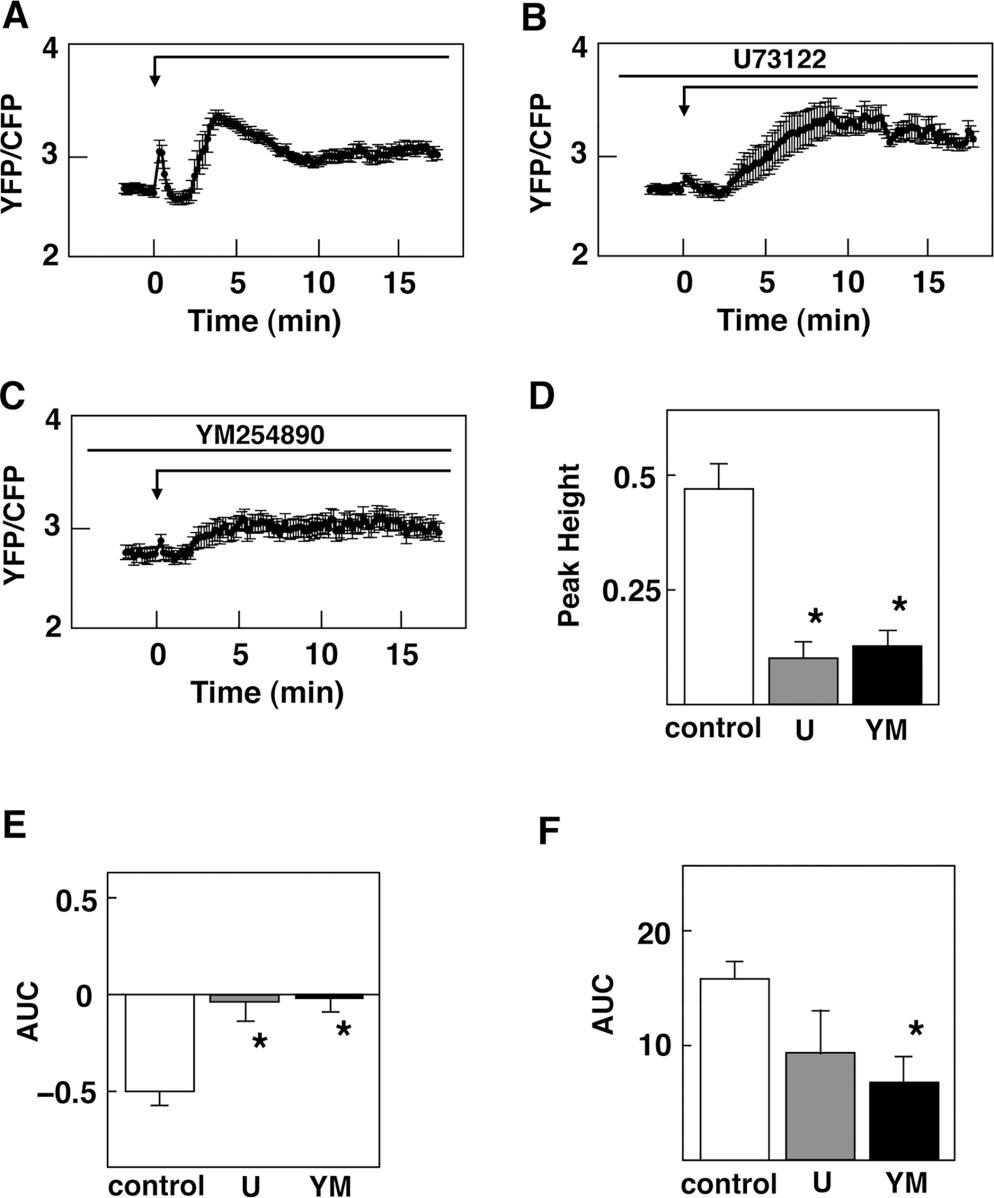
Effect of U73122 and YM254890 in Glucose-induced Changes in [Ca^2+^]_c_. A: PM-Cameleon-nano15-expressing cells were stimulated by 25 mM glucose as shown by the arrow and changes in [Ca^**2+**^]_c_ were monitored. Values are the mean ± SE for 10 determinations. B: PM-Cameleon-nano15-expressing cells were stimulated by 25 mM glucose in the presence of 10 μM U73122, which was added 10 min prior to the addition of glucose. Values are the mean ± SE for 10 determinations. C: PM-Cameleon-nano15-expressing cells were stimulated by 25 mM glucose in the presence of 10 μM YM254890, which was added 10 min prior to the addition of glucose. Values are the mean ± SE for 10 determinations. D: Experiments were carried out as shown in A, B and C. The peak height of the first rapid peak was measured. YM: YM254890, U: U73122. *: P< 0.01 vs control. E: Experiments were carried out as shown in A, B and C. AUC from 1.2 to 2.0 min was calculated. YM: YM254890, U: U73122. *: P< 0.01 vs control. F: Experiments were carried out as shown in A, B and C. AUC from 3 to 10 min was calculated. Values are the mean ± SE for 10 determinations. YM: YM254890, U: U73122. *: P< 0.05 vs control.

We first examined whether glucose evoked a rapid effect on cytoplasmic Ca^2+^. To this end, we monitored changes in [Ca^2+^]_c_ in MIN6 cells using an ultrasensitive Ca^2+^ indicator, yellow Cameleon-nano15 [34] targeted to the plasma membrane (PM-Cameleon-nano15) [32]. PM-Cameleon-nano15 enabled us to monitor subtle changes in [Ca^2+^]_c_ in these cells [32, 34]. When ambient glucose concentration was raised from 2.8 mM to 25 mM, an immediate transient elevation of [Ca^2+^]_c_ was observed. A typical response of [Ca^2+^]_c_ is shown in Fig 1A. This immediate elevation of [Ca^2+^]_c_ was observed within seconds and [Ca^2+^]_c_ peaked within 10 sec. [Ca^2+^]_c_ then rapidly decreased to a plateau level, which was lower than the basal level. [Ca^2+^]_c_ remained decreased for 1 to 5 min and then [Ca^2+^]_c_ rose abruptly. The initial rapid peak of [Ca^2+^]_c_ was observed in approximately 60% of the cells examined while the following plateau phase and the second large elevation of [Ca^2+^]_c_ were observed in almost all of the cells tested. In cells without the rapid peak of [Ca^2+^]_c_, a small hump of [Ca^2+^]_c_ was instead observed immediately after the addition of glucose (Fig 1B). [Ca^2+^]_c_ was then reduced to a plateau level, which was lower than the basal level, and remained reduced for some minutes. Then [Ca^2+^]_c_ rose abruptly and oscillatory elevation of [Ca^2+^]_c_ was observed. Note that in cells without a rapid peak of [Ca^2+^]_c_, the area under the curve (AUC) of the subsequent oscillatory elevation of [Ca^2+^]_c_ was smaller than that in cells with an initial rapid peak of [Ca^2+^]_c_. Thus AUC was significantly smaller than that in cells with a rapid peak of [Ca^2+^]_c_ (Fig 1C). The initial rapid peak of Ca^2+^ or small hump of [Ca^2+^]_c_ detected using PM-Cameleon-nano15 was never observed by monitoring [Ca^2+^]_c_ using a conventional Ca^2+^ indicator fura-2. Thus, when [Ca^2+^]_c_ was monitored by fura-2, [Ca^2+^]_c_ was decreased by the addition of 25 mM glucose, and after some minutes of interval, elevation of [Ca^2+^]_c_ was observed (data not shown). More importantly, when cells expressing PM-Cameleon-nano15 were simultaneously loaded with fura-2, the initial rapid elevation of [Ca^2+^]_c_ monitored by PM-Cameleon-nano15 was abolished (Fig 1D). In other words, loading of fura-2 abolished the initial rapid peak of [Ca^2+^]_c_. Triphasic changes in [Ca^2+^]_c_ were observed when cells were stimulated with 8.3 mM and 16.7 mM glucose (Fig 1E and 1F) and approximately 50% of the cells presented a rapid peak of [Ca^2+^]_c_. Both the first rapid peak and AUC of the second oscillatory elevation of [Ca^2+^]_c_ were increased in dose-dependent manners (Fig 1G and 1H). The first rapid peak of [Ca^2+^]_c_ was markedly inhibited by the addition of a non-specific inhibitor of phospholipase C (PLC) U73122 [35] (Fig 2A and 2B). Subsequent reduction of [Ca^2+^]_c_ was also abolished by U73122 (Fig 2B). Note that an inactive analogue U73343 was without effect (data not shown). Likewise, the first rapid peak of [Ca^2+^]_c_ and subsequent reduction of [Ca^2+^]_c_ were markedly inhibited by the addition of a G_q_ inhibitor YM254890 [36] (Fig 2C). Quantitatively, the first peak of [Ca^2+^]_c_ was significantly reduced by U73122 and YM254890 (Fig 2D). Similarly, the reduction of [Ca^2+^]_c_ was abolished by both U73122 and YM254890 (Fig 2E). It should be noted that YM254890 also significantly inhibited large oscillatory elevation of [Ca^2+^]_c_ induced by glucose (Fig 2F).

**Fig 3:**
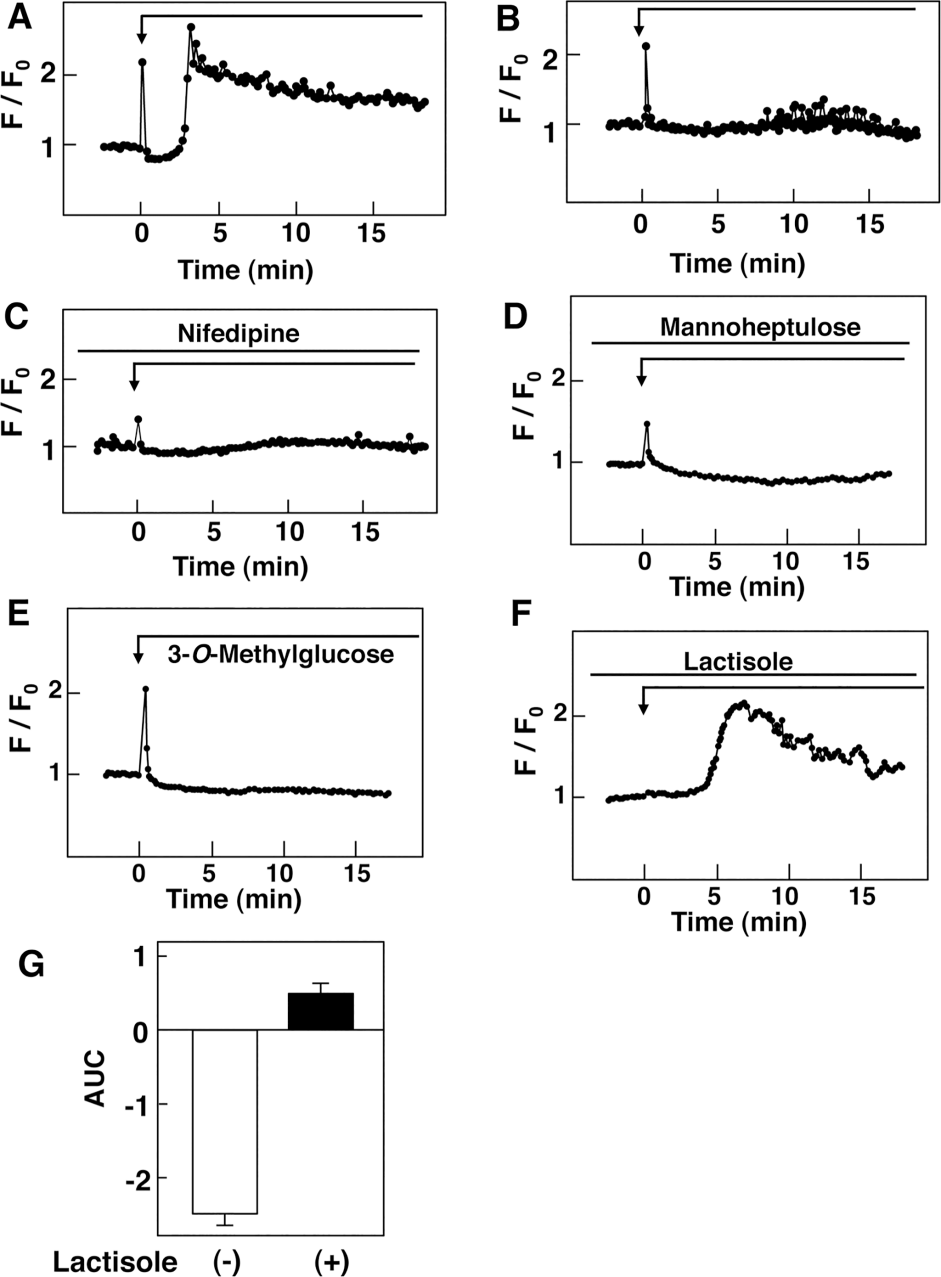
Effect of Glucose and 3-*O*-Methylglucose on [Ca^2+^]_c_ Measured by Fluo-8. A: Fluo-8-loaded cells were stimulated by 25 mM glucose and changes in [Ca^**2+**^]_c_ were measured. A typical response with a rapid peak is presented. The trace is a respresentative of those-obtained in more than 100 cells. B: Fluo-8-loaded cells were incubated in Ca^**2+**^-free HBSS and stimulated by 25 mM glucose as shown by the arrow. The trace is a representative of those obtained in more than 100 cells. C: Fluo-8-loaded cells were stimulated by 25 mM glucose in the presence of 1 μM nifedipine, which was added 10 min pror to the stimulation by glucose. Changes in [Ca^**2+**^]_c_ were monitored. The trace is a representative of those obtained in more than 100 cells. D: Fluo-8-loaded cells preincubated for 10 min with 10 mM mannoheptulose were stimulated with 25 mM glucose as indicated by the arrow. The trace is a representative of those obtained in more than 100 cells. E: Fluo-8-loaded cells were stimulated by 25 mM 3-*O*-methylglucose and changes in [Ca^**2+**^]_c_ were monitored. The trace is a representative of those obtained in more than 100 cells. F: Fluo-8-loaded cells were stimulated by 25 mM glucose in the presence of 5 mM lactisole, which was added 10 min prior to the stimulation by glucose. Changes in [Ca^**2+**^]_c_ were monitored. The trace is a representative of those obtained in more than 100 cells. G: Experiments were carried out as shown in F and AUC from 1 to 5 min was calculated. Values are the mean ± SE for 10 determinations.

We tried to detect the rapid peak of [Ca^2+^]_c_ by using conventional Ca^2+^ indicators and found that the initial rapid elevation of Ca^2+^ was detected by using fluo-8 in a fraction of the cells. As shown in Fig 3A, a rapid elevation of [Ca^2+^]_c_ induced by 25 mM glucose was observed in approximately 40% of the fluo-8-loaded cells. In these cells, triphasic changes in [Ca^2+^]_c_ were qualitatively similar to those observed by using PM-Cameleon-nano15. When extracellular Ca^2+^ was removed, glucose-mediated initial peak of [Ca^2+^]_c_ was observed but the large elevation of [Ca^2+^]_c_ observed several minutes later was markedly inhibited (Fig 3B). Similar results were obtained when cells were stimulated by 25 mM glucose in the presence of 1 μM nifedipine, an inhibitor of L-type voltage-gated calcium channel (Fig 3C). Note that the rapid peak of [Ca^2+^]_c_ was smaller compared to Fig 3A. We then assessed the involvement of the glucose-sensing receptor in glucose-induced changes in [Ca^2+^]_c_. We first blocked glucose metabolism by adding mannoheptulose, an inhibitor of glucokinase. In the presence of 10 μM mannoheptulose, glucose induced a rapid elevation of [Ca^2+^]_c_ and subsequent sustained reduction of [Ca^2+^]_c_. The magnitude of the first peak of [Ca^2+^]_c_ was smaller compared to that in the absence of mannoheptulose. A large elevation of [Ca^2+^]_c_ observed after a lag period of some minutes was blocked (Fig 3D). Similar results were obtained by adding a nonmetabolizable glucose analogue, 3-*O*-methylglucose, instead of glucose. Thus, 3-*O*-methylglucose induced a rapid elevation of [Ca^2+^]_c_ and subsequent reduction of [Ca^2+^]_c_. However, a large elevation of [Ca^2+^]_c_ after a lag period of some minutes was not observed (Fig 3E). When the glucose-sensing receptor was blocked by adding lactisole, an inhibitor of T1R3 [37], glucose did not induce a rapid elevation of [Ca^2+^]_c_ (Fig 3F). Subsequent reduction of [Ca^2+^]_c_ was not observed. In contrast, elevation of [Ca^2+^]_c_ was observed several minutes later. Quantitatively, the decrease in [Ca^2+^]_c_ was abolished by lactisole (Fig 3G).

### Effect of Glucose on cAMP

**Fig 4:**
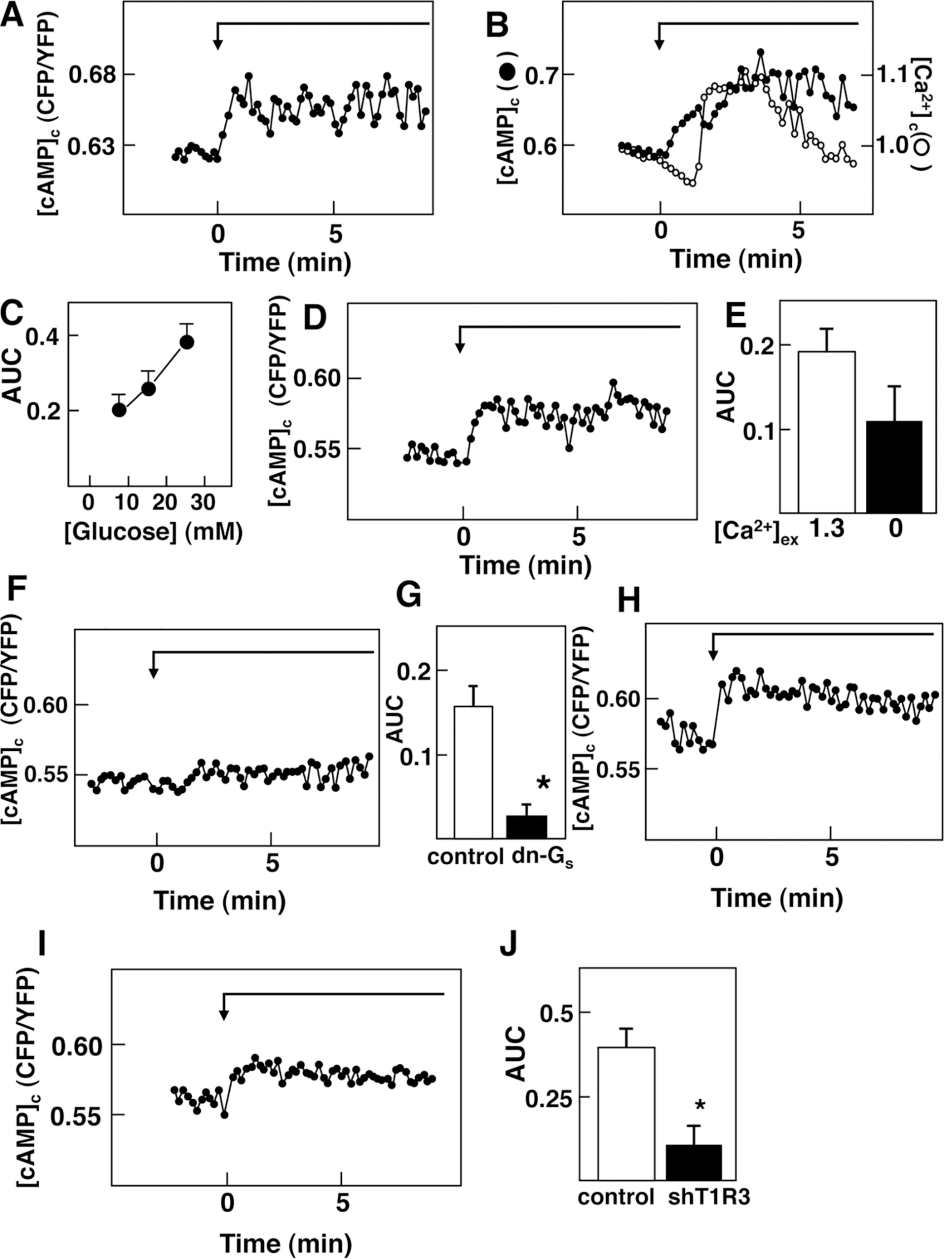
Effect of Glucose on [cAMP]_c_ in MIN6 Cells. A: Epac1-camps-expressing cells were stimulated by 25 mM glucose and changes in [cAMP]_c_ were monitored. The result is a representative of those obtained in more than 100 cells. B: Epac1-camps-expressing cells loaded with fura-2 were stimulated by 25 mM glucose and changes in [cAMP]_c_ (●) and [Ca^**2+**^]_c_ (○) were monitored stimutaneously. The result is a representative of those obtained in 25 cells. C: Epac1-camp-expressing cells were stimulated by 8.3, 16.7 and 25 mM glucose and AUC of the [cAMP]_c_ response from 0 to 8 min was calculated. Values are the mean ± SE for 8 determinations. D: Epac1-camps-expressing cells were incubated in Ca^**2+**^-free HBSS containing 0.2 mM EGTA and stimulated by 25 mM glucose. Changes in [cAMP]_c_ were monitored. The result is a representative of those obtained in 50 cells. E: Experiments were carried out as shown in A and D. AUC from 0 to 5 min was calculated. [Ca^**2+**^]_ex_: concentration of extracellular Ca^**2+**^. F: Cells expressing Epac1-camps and dominant-negative G_s_ were stimulated by 25 mM glucose and changes in [cAMP]_c_ were monitored. The result is a representative of those obtained in 50 cells. G: Experiments were carried out as shown in A and F and AUC from 0 to 5 min was calculated. Values are the mean ± SE for 10 determinations. dn-G_s_: dominant-negative G_s_. *: P< 0.01 vs control. H: Epac1-camps-expressing cells were stimulated by 25 mM glucose in the presence of 10 mM mannoheptulose. The result is a representative of those obtained in more than 25 cells. I: Epac1-camps-expressing cells were stimulated by 25 mM 3-*O*-methylglucose and changes in [cAMP]_c_ were monitored. The result is a representative of those obtained in 20 cells. J: T1R3 was knocked down by using shT1R3. Control cells and T1R3-knocked down cells were stimulated by 25 mM glucose and changes in [cAMP]_c_ were monitored. AUC from 0.3 to 2.2 min was calculated. Values are the mean ± SE for five determinations. *: P< 0.05 vs control.

We next monitored changes in [cAMP]_c_ induced by glucose using a cAMP indicator Epac1-camps [30]. When Epac1-camps-expressing MIN6 cells were stimulated by 25 mM glucose, a rapid and sustained elevation of [cAMP]_c_ was observed (Fig 4A). Elevation of [cAMP]_c_ was observed within 10 sec of the addition of glucose, and [cAMP]_c_ reached a plateau level approximately 1 min after the addition of glucose. When changes in [cAMP]_c_ and [Ca^2+^]_c_ were monitored simultaneously in Epac1-camps-expressing cells loaded with fura-2, glucose induced reduction of [Ca^2+^]_c_ and the elevation of [cAMP]_c_ were observed even in the same period (Fig 4B), indicating that [cAMP]_c_ was increased when [Ca^2+^]_c_ was reduced. Glucose-induced elevation of [cAMP]_c_ was observed at a concentration of 8.3 mM and was increased in a dose-dependent manner (Fig 4C). Elevation of [cAMP]_c_ was observed even in the absence of extracellular Ca^2+^ (Fig 4D). AUC of the [cAMP]_c_ response in the absence of extracellular calcium was not changed significantly compared to that in the presence of extracellular calcium (Fig 4E). On the other hand, the elevation of [cAMP]_c_ was abolished by introducing dominant-negative G_s_ (Fig 4F and 4G). Note that dominant-negative G_s_ did not affect the elevation of [cAMP]_c_ induced by depolarizing concentration of KCl (data not shown). This rapid elevation of [cAMP]_c_ was observed in the presence of mannoheptulose (Fig 4H). Furthermore, 3-*O*-methylglucose, a nonmetabolizable analogue of glucose, essentially reproduced the effect of glucose (Fig 4I). When T1R3 was knocked down, glucose-induced elevation of [cAMP]_c_ was markedly inhibited (Fig 4J).

### Effect of Glucose on Activation of Protein Kinase C

**Fig 5:**
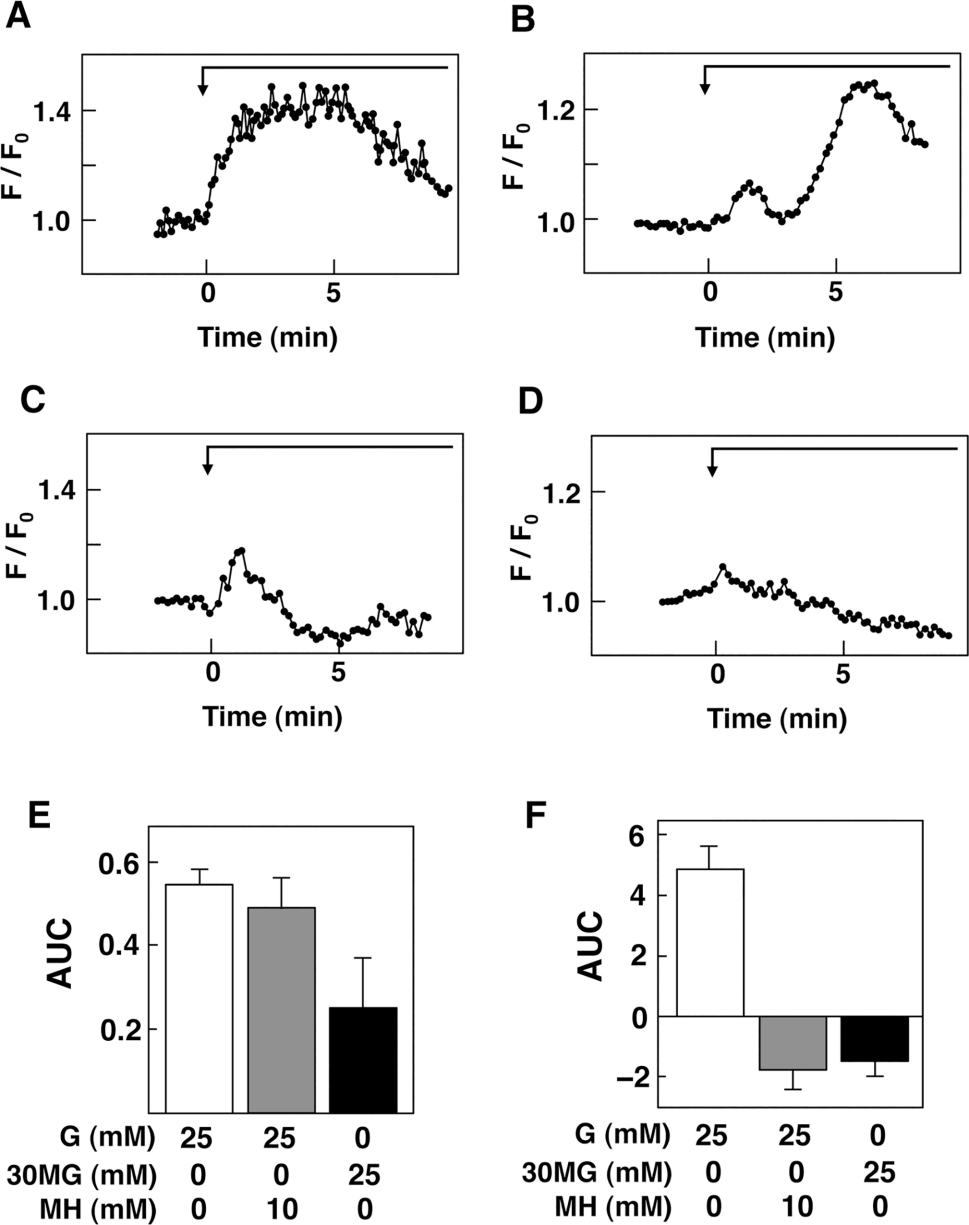
Effect of Glucose on DAG and MARCKS in MIN6 Cells. A: C1_2_-mRFP-expressing cells were stimulated by 25 mM glucose and changes in DAG were monitored. The result is a representative of those obtained in 20 cells. B: MARCKS-GFP-expressing cells were stimulated by 25 mM glucose and changes in MARCKS-GFP in cytoplasm were monitored. The result is a representative of those obtained in more than 100 cells. C: MARCKS-GFP-expressing cells were stimulated by 25 mM glucose in the presence of 10 mM mannoheptulose (MH) and changes in MARCKS-GFP in cytoplasm was monitored. The trace is a representative of those obtained in 20 cells. D: MARCKS-GFP-expressing cells were stimulated by 25 mM 3-*O*-methylglucose and changes in MARCKS-GFP in cytoplasm were monitored. E, F: Experiments were carried out as shown in A, B and C and AUC from 0.3 to 3 min (E) and from 3.3 to 8 min (F) was calculated. G: glucose, 3OMG: 3-*O*-methylglucose, MH: mannoheptulose.

We then measured changes in DAG. As shown in Fig 5A, the addition of 25 mM glucose induced a rapid and monophasic increase in DAG. Elevation of DAG was detected within 10 sec and the peak was observed around 3 min of stimulation by glucose. We then monitored activation of protein kinase C by monitoring phosphorylation state of MARCKS in cytosol, a substrate for protein kinase C that translocates from the plasma membrane to cytosol upon phosphorylation [31]. As shown in Fig 5B, the addition of 25 mM glucose induced biphasic elevation of phosphorylated MARCKS in cytosol. An initial increase was observed within 20 sec and peaked at 2 to 3 min after the addition of glucose. The second larger peak of phosphorylated MARCKS in cytosol was observed 5 to 7 min after the addition of glucose. When mannoheptulose was included to block metabolism of glucose, the first peak was observed whereas the second peak was abolished (Fig 5C). Addition of 3-*O*-methylglucose, a nonmetabolizable glucose analogue, evoked the first peak whereas the second peak was not observed and the level of phosphorylated MARCKS in cytosol was decreased gradually (Fig 5D). Results of the quantitative analyses of the effects of mannoheptulose and 3-*O*-methylglucose are shown in Fig 5E and 5F.

### Effect of Glucose on Cytoplasmic Ca^2+^ in β-cells Obtained From Normal and T1R3-knockout Mice

**Fig 6:**
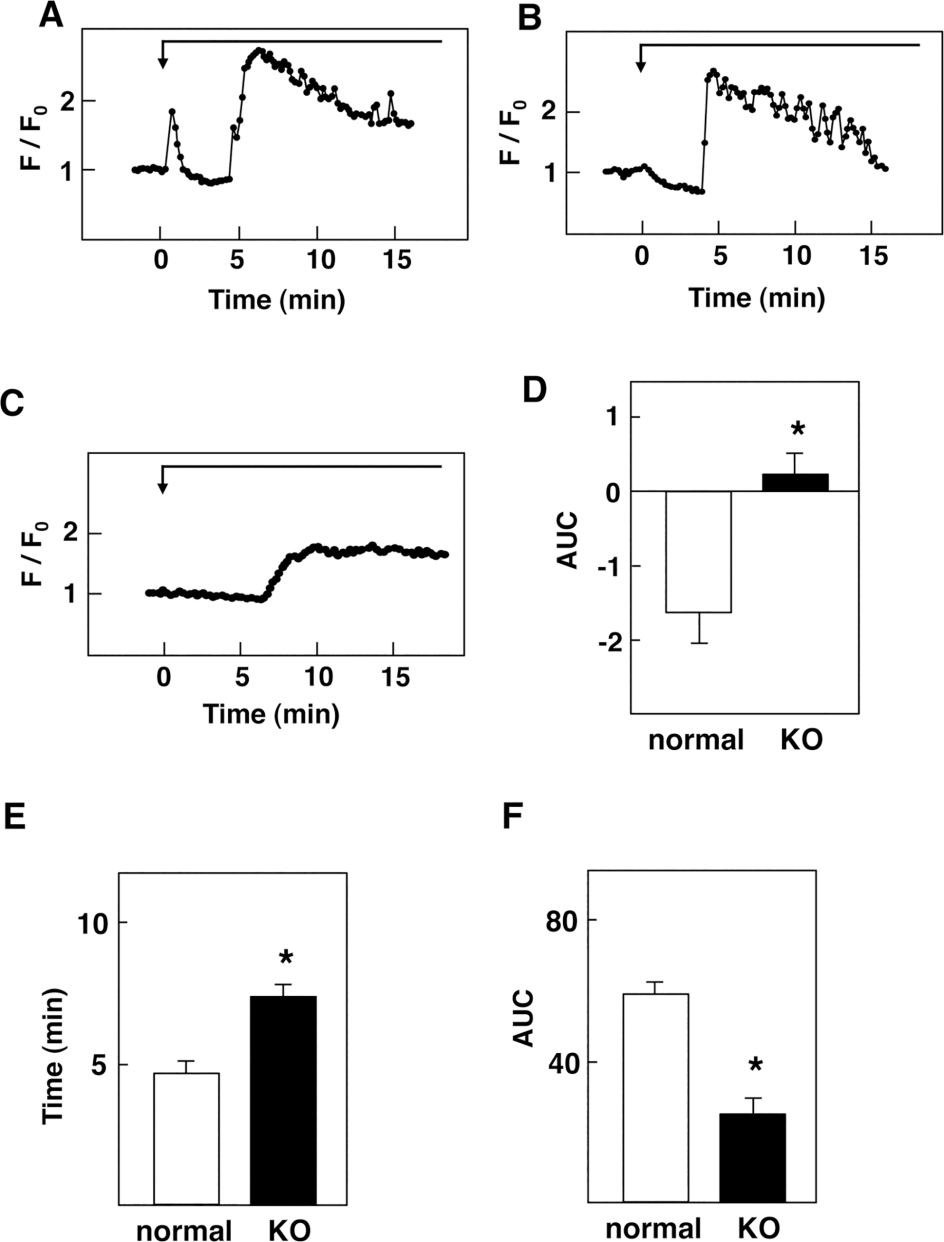
Effect of [Ca^2+^]_c_ in β-cells Obtained from Normal and T1R3 Knocked Mice. A, B: Isolated β-cells obtained from normal mouse were stimulated by 16.7 mM glucose and changes in [Ca^2+^]_c_ were monitored by using fluo-8. Two types of responses, with (A) or without (B) a rapid peak of [Ca^2+^]_c_, were obtained. C: Isolated β-cells obtained from T1R3 knockout mouse were stimulated by 16.7 mM glucose and changes in [Ca^2+^]_c_ were monitored by using fluo-8. The result is a representative of those obtained in more than 100 cells. D: Experiments were carried out as shown in A, B and C and AUC from 2 to 5 min were calculated. Values are the mean ± SE for 20 determinations. *: P< 0.01 vs normal. E: Experiments were carried out as shown in A, B and C. The latent period until the initiation of oscillatory elevation of [Ca^2+^]_c_ was measured. Values are the mean ± SE for 20 determinations. *: P< 0.05 vs normal. F: Experiments were carried out as shown A, B and C. AUC from 5 to 15 min was calculated. Values are the mean ± SE for 20 determinations. *: P< 0.01 vs normal.

**Fig 7:**
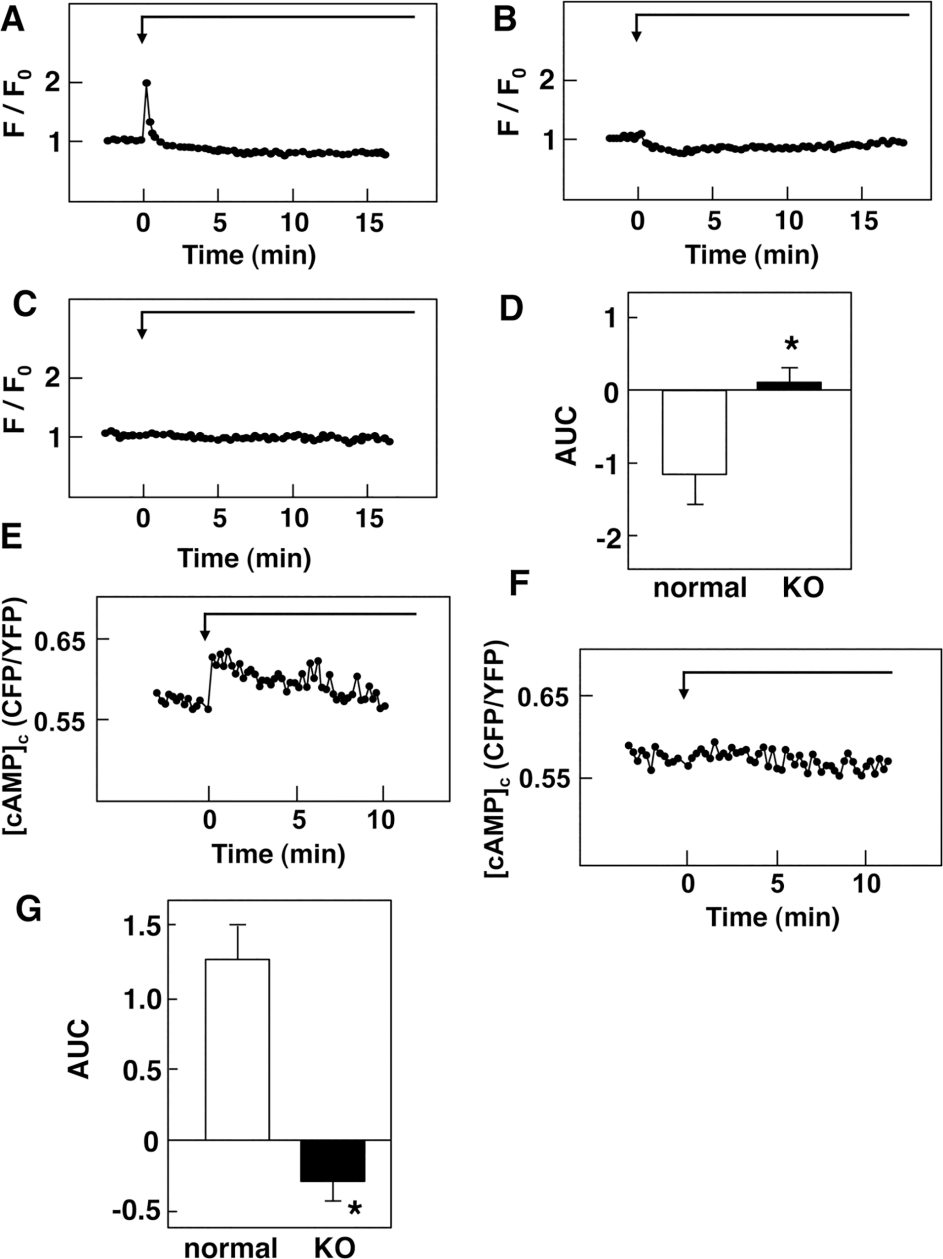
Effect of 3-*O*-Methylglucose on [Ca^2+^]_c_ and [cAMP]_c_ in β-cells Obtained from Normal and T1R3 Knocked Mice. A, B: Isolated β-cells obtained from normal mice were stimulated by 16.7 mM 3-*O*-methylglucose and changes in [Ca^2+^]_c_ were measured by using fluo-8. Two types of responses, with (A) or without (B) a rapid peak of [Ca^2+^]_c_, were obtained. C: Isolated β-cells obtained from T1R3 knocked mice were stimulated by 16.7 mM 3-*O*-methylglucose and changes in [Ca^2+^]_c_ were monitored by using fluo-8. D: Experiments were carried out as shown in A, B and C and AUC from 1 to 5 min were calculated. Note that each value was subtracted by the basal value, which was the mean of the prestimulated values. Values are the mean ± SE for 20 determinations. *: P< 0.05 vs control. E, F: Epac1-camps-expressing β-cells obtained from normal (E) and T1R3 knockout (F) mice were stimulated by 16.7 mM glucose and changes in [cAMP]_c_ were monitored. The results are the representative of those obtained in more than 20 cells. G: Experiments were carried out as shown in E and F and AUC from 0 to 5 min was calculated. Values are the mean ± SE for 20 cells. *: P< 0.01 vs control.

To further confirm the role of the glucose-sensing receptor, we studied the effect of glucose on [Ca^2+^]_c_ in β-cells obtained from normal and T1R3-knockout mice by using a Ca^2+^ indicator fluo-8. In normal β-cells, a high concentration of glucose induced a rapid peak of [Ca^2+^]_c_ in approximately 45% of the cells examined. After the sharp peak of [Ca^2+^]_c_, [Ca^2+^]_c_ then reduced to a value below the basal level. After a lag period of approximately 5 min, a large elevation of [Ca^2+^]_c_ was observed (Fig 6A). In cells without the rapid peak of [Ca^2+^]_c_, glucose induced a gradual decrease in [Ca^2+^]_c_, which was followed by an abrupt elevation of [Ca^2+^]_c_ (Fig 6B). [Ca^2+^]_c_ remained elevated for more than 10 min. In T1R3-null β-cells, the glucose-induced rapid peak of [Ca^2+^]_c_ was never observed. Moreover, a high concentration of glucose did not cause a decrease in [Ca^2+^]_c_. After a lag period of approximately 7 min, an increase in [Ca^2+^]_c_ was observed (Fig 6C). Compared to that in normal β-cells, elevation of [Ca^2+^]_c_ was blunted. Quantitatively, initial decrease in [Ca^2+^]_c_ induced by glucose was abolished in T1R3-null β-cells (Fig 6D). The onset of the large elevation of [Ca^2+^]_c_ was significantly delayed in T1R3-null β-cells (Fig 6E). In addition, AUC of the elevation of [Ca^2+^]_c_ induced by high concentration of glucose was markedly reduced in T1R3-null β-cells (Fig 6F). We then examined the effect of 3-*O*-methylglucose on [Ca^2+^]_c_ in normal β-cells. As shown in Fig 7A, addition of 3-*O*-methylglucose induced a rapid elevation of [Ca^2+^]_c_, which was followed by a sustained decrease in [Ca^2+^]_c_ in normal β-cells. The initial rapid peak of [Ca^2+^]_c_ was observed in approximately 40% of the cells. In the rest of cells, the initial rapid peak was not observed and [Ca^2+^]_c_ was decreased in response to 3-*O*-methylglucose (Fig 7B). In contrast, 3-*O*-methylglucose did not induce a rapid peak of [Ca^2+^]_c_ nor a sustained decrease in [Ca^2+^]_c_ in T1R3-null β-cells (Fig 7C). Quantitatively, sustained decrease in [Ca^2+^]_c_ induced by 3-*O*-methylglucose was abolished in T1R3-null β-cells (Fig 7D).

We then measured changes in [cAMP]_c_ induced by glucose in normal β-cells. As shown in Fig 7E, glucose evoked a rapid and sustained elevation of [cAMP]_c_. Elevation of [cAMP]_c_ was observed within 10 sec. In contrast, glucose did not increase [cAMP]_c_ in T1R3-null β-cells (Fig 7F). Quantitatively, elevation of [cAMP]_c_ induced by glucose was markedly reduced in T1R3-null β-cells (Fig 7G).

## Discussion

The present study was conducted to detect receptor-mediated rapid signals evoked by glucose in pancreatic β-cells. Using an ultrasensitive Ca^2+^ indicator Cameleon-nano15 [34] targeted to the plasma membrane [32], we could detect subtle changes in [Ca^2+^]_c_ in β-cells. As shown in Fig 1A, glucose induced an immediate transient increase in [Ca^2+^]_c_ in β-cells. This rapid elevation of [Ca^2+^]_c_ has never been reported to date. This is because the elevation of [Ca^2+^]_c_ was rapid and subtle, and was only detected by using a very sensitive indicator PM-Cameleon-nano15 but not by conventional Ca^2+^ indicators such as fura-2. As demonstrated in Fig 1D, loading of fura-2 abolished this rapid Ca^2+^ signal detected by PM-Cameleon-nano15. The chelating action of fura-2 may have buffered the tiny changes in [Ca^2+^]_c_. In any case, a sensitive method enabled us to detect the immediate Ca^2+^ signal induced by high concentration of glucose. It should be noted that, when we monitored [Ca^2+^]_c_ by using a Ca^2+^ indicator with stronger fluorescence fluo-8, we could detect the rapid peak of [Ca^2+^]_c_ in a fraction of the cells (Fig 1E). This elevation is due at least partly to release of Ca^2+^ from an intracellular pool(s) since the rapid elevation of [Ca^2+^]_c_ was observed in the absence of extracellular Ca^2+^. Since the rapid elevation of [Ca^2+^]_c_ was inhibited by an inhibitor of PLC and an inhibitor of G_q_, it may be due to a release of calcium from ER caused by inositol trisphosphate. This rapid elevation of [Ca^2+^]_c_ was observed even if glucose metabolism was blocked by mannoheptulose, indicating that it is not dependent on glucose metabolism. Instead, the rapid elevation was blocked by lactisole, an inhibitor of T1R3 [37]. Furthermore, the rapid peak of [Ca^2+^]_c_ was never observed in T1R3-null β-cells. The rapid peak of [Ca^2+^]_c_ evoked by glucose is therefore caused by activation of the glucose-sensing receptor. In agreement with this notion, activation of the receptor by an administration of nonmetabolizable glucose analogue 3-*O*-methylglucose reproduced the rapid elevation of [Ca^2+^]_c_. Collectively, the rapid elevation of [Ca^2+^]_c_ is a signal due to activation of the glucose-sensing receptor. It should be mentioned that the rapid elevation of [Ca^2+^]_c_ induced by glucose is different from the elevation of [Ca^2+^]_c_ induced by typical calcium-mobilizing agonists, for example, carbachol [24] in many respect. Although both glucose and carbachol may activate PLC and elevate [Ca^2+^]_c_, [Ca^2+^]_c_ response induced by glucose is subtle and is not detected by fura-2 whereas that induced by carbachol is easily detectable by fura-2 [24]. Also, glucose-induced rapid elevation of [Ca^2+^]_c_ was only transient and [Ca^2+^]_c_ decayes quickly. In addition, glucose-induced rapid elevation of [Ca^2+^]_c_ is followed by a sustained decrease in [Ca^2+^]_c_ while that induced by carbachol is not. Presumably, glucose and carbachol activate PLC differently and elicit slightly different [Ca^2+^]_c_ signals.

The present results indicate that there are two populations of β-cells with regard to the [Ca^2+^]_c_ response to glucose: roughly half of the cells presented a rapid peak of [Ca^2+^]_c_ induced by glucose whereas rest of them did not. Difference in the expression levels of T1R3 would explain the different patterns of [Ca^2+^]_c_ response in two populations. However, when we measured the expression of T1R3 in pancreatic islets [25] and in MIN6 cells [24], the expression of T1R3 was not so different among β-cells. Presumably, difference in the expression of other signaling molecules would be responsible for different properties found in two populations.

Previous studies have shown that glucose induced reduction of [Ca^2+^]_c_, which is due largely to sequestration of Ca^2+^ into ER through activation of SERCA [9–13]. Nonetheless, the mechanism by which glucose promotes sequestration of Ca^2+^ into ER is not certain [38]. Indeed, inhibition of the glucose-sensing receptor by lactisole [37] abolished the reduction of [Ca^2+^]_c_ induced by glucose (Fig 2C). Similarly, reduction of [Ca^2+^]_c_ was abolished in β-cells obtained from T1R3 knockout mice. Moreover, the addition of 3-*O*-methylglucose reproduced the reduction of [Ca^2+^]_c_ induced by glucose. These results strongly suggest that the reduction of [Ca^2+^]_c_ induced by glucose is mediated by the glucose-sensing receptor. At present, the mechanism by which this receptor stimulates sequestration of Ca^2+^ to ER via activation of SERCA is not totally certain. In this regard, reduction of [Ca^2+^]_c_ was abolished by a G_q_ inhibitor and an inhibitor of PLC. The reduction of [Ca^2+^]_c_ may be linked to receptor-mediated activation of PLC. When we expressed T1R3 in HEK293 cells, the addition of 25 mM glucose induced a rapid elevation of [Ca^2+^]_c_ followed by sustained reduction of [Ca^2+^]_c_ (unpublished observation). Reduction of [Ca^2+^]_c_ following a rapid elevation of [Ca^2+^]_c_ may result from a unique property of the glucose-sensing receptor. In addition, activation of T1R3 leads to reduction of [Ca^2+^]_c_ at least in some occasions. In human GLP-1-secreting cells, stimulation of the glucose-sensing receptor by acesulfame-K persistently reduced [Ca^2+^]_c_ [39]. The reduction of [Ca^2+^]_c_ is due to activation of the plasma membrane Ca^2+^ pump (PMCA) [39]. Although the precise mechanism by which the receptor activates PMCA is still unclear, this is another example showing that binding of a ligand to the glucose-sensing receptor causes activation of the Ca^2+^ pump leading to sustained reduction of [Ca^2+^]_c_. In β-cells, activation of the receptor by glucose may cause activation of SERCA and reduces [Ca^2+^]_c_. Further study is obviously needed to identify the mechanism of activation of SERCA by the glucose-sensing receptor. In this regard, it is possible that increase in subplasma membrain Ca^2+^ activates SERCA and promotes uptake of Ca^2+^ to ER. Alternately, since elevation of cAMP stimulates sequestration of Ca^2+^ to ER [40], it is possible that cAMP produced by the receptor activation of G_s_ would cause sequestration of Ca^2+^ to ER. Further studies are needed to identify the mechanism. It should be mentioned that the reduction of [Ca^2+^]_c_ induced by glucose is also dependent on glucose metabolism to some extent. This is because SERCA is an ATP-requiring enzyme and glucose elevates intracellular ATP within a minute [27]. Elevated ATP would facilitate uptake of Ca^2+^ to ER.

In the present study, we monitored the glucose effect on [cAMP]_c_ by paying particular attention to a rapid action of glucose. The results obtained in MIN6 cells and mouse β-cells show that glucose induces an immediate elevation of [cAMP]_c_. Glucose-induced elevation of [cAMP]_c_ preceded a large oscillatory elevation of [Ca^2+^]_c_ and was observed even in the absence of extracellular calcium. These results suggest that glucose-mediated elevation of [cAMP]_c_ is at least partly independent of elevation of [Ca^2+^]_c_. We have previously shown that the glucose-sensing receptor causes elevation of [cAMP]_c_ by a G_s_-dependent mechanism [29]. In addition, activation of the glucose-sensing receptor by 3-*O*-methylglucose increases [cAMP]_c_ (Fig 4I). Our results are in agreement with the report by Kim et al. [22] and Fridlyand et al. [41]. However, our results are not consistent with previous reports showing that elevation of [cAMP]_c_ is secondary to the elevation of [Ca^2+^]_c_ [18]. The reason for the discrepancy is not totally certain. However, our measurement was focused on the early response of [cAMP]_c_ in β-cells. It is likely that at least rapid elevation of [cAMP]_c_ is caused by receptor-mediated activation of G_s_.

In the present study, we also showed that glucose induced rapid and long-lasting activation of protein kinase C by showing that glucose induces biphasic increase in phosphorylated MARCKS in cytosol. It has been generally thought that glucose activates protein kinase by elevating [Ca^2+^]_c_ [42]. In β-cells, elevation of [Ca^2+^]_c_ would activate calcium-dependent PLC leading to generation of DAG [43]. Alternately, high concentration of glucose stimulates *de novo* synthesis of DAG by supplying a substrate [44]. In any event, it has been thought that activation of protein kinase C is dependent on glucose metabolism and is rather slow in onset. As shown in Fig 3B, however, phosphorylation of MARCKS was detected within 10 seconds and increase in DAG was also detected within 10 sec. Furthermore, at least the first phase of elevation of phosphorylated MARCKS was independent of glucose metabolism and was reproduced by a nonmetabolizable glucose analogue. Our previous results show that the glucose-sensing receptor is coupled to the PLC-calcium messenger system [24]. Collectively, a rapid activation of protein kinase C may be independent of glucose metabolism and is caused by receptor-mediated activation of PLC.

The present study demonstrates for the first time that glucose evokes rapid intracellular signals, Ca^2+^ and cAMP, in pancreatic β-cells. These actions are independent of the glucose metabolism and are mediated by the glucose-sensing receptor. Importantly, inhibition of the receptor or deletion of the T1R3 gene attenuates glucose-induced insulin secretion [37, 45]. Specifically, insulin secretion is markedly delayed and blunted in β-cells obtained from T1R3 knockout mice [45]. Hence, these receptor-mediated rapid signals are critical for the glucose action in β-cells. Collectively, the action of glucose is not solely dependent on its metabolism [46]. The glucose-sensing receptor generates rapid signals and, by priming the metabolic pathway, enhances the pathway dependent on the glucose metabolism [26]. Our results are different from the postulate by Kyriazis et al. [47] that the sweet taste receptor inhibits basal secretion of insulin. As we mentioned previously [37], their conclusion is solely dependent on the results obtained by using a T1R3 inhibitor lactisole. Moreover, their data may result from an inadequate use of lactisole [37]. In fact, their results obtained by lactisole are even contradictory to their own results [47, 48]. More importantly, glucose-induced insulin secretion is obviously attenuated in T1R3-null β-cells both in vitro [37, 45] and in vivo [49], it is certain that T1R3 is involved in the action of glucose in pancreatic β-cells.

In summary, we identified the intracellular signals evoked by glucose via the activation of the glucose-sensing receptor. These rapid signals may be important for the priming of the metabolic pathway [26, 27] and for rapid secretion of insulin [37, 45].

## References

[1] Rasmussen H, Zawalich KC, Ganeson S, Calle R, Zawalich WS. Physiology and pathophysiology of insulin secretion. Diab Care. 1990; 13: 655–666. 10.2337/diacare.13.6.655 2192849

[2] Henquin JC. Regulation of insulin secretion: a matter of phase control and amplitude modulation. Diabetologia. 2009; 52: 739–751. 10.1007/s00125-009-1314-y 19288076

[3] Rorsman P. The pancreatic beta-cells as a fuel sensor: an electrophysiologist’s viewpoint. Diabetologia. 1997; 40: 487–498. 9165215 10.1007/s001250050706

[4] Henquin JC, Meissner HP. Significance of ionic fluxes and changes in membrane potential for stimulus-secretion coupling in pancreatic B-cells. Experientia. 1984; 40: 1043–1052. 6386515 10.1007/BF01971450

[5] Ashcroft FM, Rorsman P. Electrophysiology of the pancreatic B-cell. Prog Biophys Mol Biol. 1989; 54: 87–143. 2484976 10.1016/0079-6107(89)90013-8

[6] Dean PM, Mathew EK. Electrical activity in pancreatic islet cells. Nature. 1968; 219: 389–390. 4873864 10.1038/219389a0

[7] Cook DL, Hales CN. Intracellular ATP directly blocks K^+^-channels in pancreatic B-cells. Nature. 1984; 312: 271–273. 6090930 10.1038/311271a0

[8] Rorsman P, Trube G. Glucose-dependent K^+^-channels in pancreatic β-cells are regulated by intracellular ATP. Pfluger Arch. 1985; 405: 305–309. 10.1007/BF00595682 2417189

[9] Rorsman P, Abrahamsson H, Gylfe E, Hellman B. Dual effects of glucose on the cytosolic Ca^2+^ activity of mouse pancreatic β-cells. FEBS Lett. 1984; 170: 196–200. 6373371 10.1016/0014-5793(84)81398-8

[10] Grapengiesser E, Gylfe E, Hellman B. Glucose induced oscillation of cytoplasmic Ca^2+^ in the pancreatic β-cell. Biochem Biophys Res Commun. 1988; 151: 1299–1394. 3281672 10.1016/s0006-291x(88)80503-5

[11] Yada T, Kake M, Tanaka H. Single pancreatic β-cells from neonatal rats exhibits as initial decrease and subsequent increase in cytosolic free Ca^2+^ in response to glucose. Cell Calcium. 1992; 13: 69–76. 1540989 10.1016/0143-4160(92)90031-m

[12] Roe MW, Merts RJ, Lancaster ME, Worley JF, Dukes ID. Thapsigargin inhibits the glucose-induced decrease of intracellular Ca^2+^ in mouse islets of Langerhans. Am J Physiol. 1994; 266: E852–E862. 8023914 10.1152/ajpendo.1994.266.6.E852

[13] Chow RH, Lund PE, Loser S, Panten U, Gylfe E. Coincidence of early glucose-induced depolarization with lowering of cytoplasmic Ca^2+^ in mouse pancreatic β-cells. J Physiol. 1995; 485: 607–617. 7562604 10.1113/jphysiol.1995.sp020756 PMC1158031

[14] Tengholm A, Hellman B, Gylfe E. The endoplasmic reticulum is a glucose-modulated high-affinity sink for Ca^2+^ in mouse pancreatic β-cells. J Physiol. 2001; 530: 533–540. 11158282 10.1111/j.1469-7793.2001.0533k.x PMC2278424

[15] Ravier MA, Daro D, Rama LP, Jonas JC, Cheng-Xue R, Schuit FC, et al Mechanism of control of the free Ca^2+^ concentration in the endoplasmic reticulum of mouse pancreatic β-cells: interplay with cell metabolism and [Ca^2+^]_c_ and role of SERCA2b and SERCA3. Diabetes. 2011; 60: 2533–2545. 10.2337/db10-1543 21885870 PMC3178295

[16] Charles M, Franska R, Schmid F, Forshan P, Grodsky G. Adenosine 3’, 5’-monophosphate in pancreatic islets: glucose-induced insulin release. Science. 1973; 179: 569–571. 4346825 10.1126/science.179.4073.569

[17] Gill V, Cerasi E. Activation of adenyl cyclase in pancreatic islets of the rat. FEBS Lett. 1973; 33: 311–314. 4353994 10.1016/0014-5793(73)80218-2

[18] Landa LJ, Harbeck M, Kaihara K, Chepurny O, Kitiphongspattana K, Graf O, et al Interplay of Ca^2+^ and cAMP signaling in the insulin-secreting MIN6 beta-cell line. J Biol Chem. 2005; 280: 31294–31302. 15987680 10.1074/jbc.M505657200 PMC3508785

[19] Furman B, Ong WK, Pyne NJ. Cyclic AMP signaling in pancreatic islets. Adv Exp Med Biol. 2010; 654: 281–304. 10.1007/978-90-481-3271-3_13 20217503

[20] Leech C, Castonguay M, Harbener J. Expression of adenylyl cyclase subtypes in pancreatic beta-cells. Biochem Biophys Res Commun. 1999; 254: 703–706. 9920805 10.1006/bbrc.1998.9906

[21] Delmeire D, Flamez D, Hinke S, Cali J, Pipeleers D, Schuit F. Type VIII adenylyl cyclase in rat beta cells: coincidence signal detector/generator for glucose and GLP-1. Diabetologia. 2003; 46: 1383–1393. 13680124 10.1007/s00125-003-1203-8

[22] Kim J, Roberts C, Berg S, Caicedo A, Roper S, Chaudhari N. Imaging cyclic AMP changes in pancreatic islets of transgenic reporter mouse. PLoS ONE. 2008; 3: e2127 10.1371/journal.pone.0002127 18461145 PMC2330161

[23] Nelson G, Hoon MA, Chandrasekar J, Zhang Y, Ryba NJP, Zuker C. Mammalian sweet taste receptors. Cell. 2001; 106: 381–390. 11509186 10.1016/s0092-8674(01)00451-2

[24] Nakagawa Y, Nagasawa M, Yamada S, Hara A, Mogami H, Nikolaev VO, et al Sweet taste receptor expressed in pancreatic β-cells activates the calcium and cyclic AMP signaling systems and stimulates insulin secretion. PLoS ONE. 2009; 4: e5106 10.1371/journal.pone.0005106 19352508 PMC2663034

[25] Medina A, Nakagawa Y, Ma J, Li L, Hamano K, Akimoto T, et al Expression of the glucose-sensing receptor T1R3 in pancreatic islet: changes in the expression levels in various nutritional and metabolic states. Endocr J. 2014; 61: 797–895. 24898279 10.1507/endocrj.ej14-0221

[26] Kojima I, Nakagawa Y, Ohtsu Y, Hamano K, Medina J, Nagasawa M. Return of the glucoreceptor: glucose activates the glucose-sensing receptor T1R3 and facifitates metabolism in pancreatic β-cells. J Diab Invest. 2015; 6: 256–263. 10.1111/jdi.12304 PMC4420555 25969708

[27] Nakagawa Y, Ohtsu Y, Nagasawa M, Shibata H, Kojima I. Glucose promotes its own metabolism by acting on the cell-surface glucose-sensing receptor T1R3. Endocr J. 2014; 61: 119–131. 24200979 10.1507/endocrj.ej13-0431

[28] Miyazaki J, Yamato E, Ikegami H, Asano T, Shibasaki Y, Oka Y, et al Establishment of a pancreatic beta cell line that retains glucose-inducible insulin secretion: special reference to expression of glucose transporter isoforms. Endocrinology. 1990; 127: 126–132. 2163307 10.1210/endo-127-1-126

[29] Nakagawa Y, Nagasawa M, Mogami H, Lohse M, Ninomiya Y, Kojima I. Multimodal function of the sweet taste receptor expressed in pancreatic β-cells: generation of diverse patterns intracellular signals by sweet agonists. Endocr J. 2013; 60: 1191–1206. 23933592 10.1507/endocrj.ej13-0282

[30] Nikolaev DV, Bunemann M, Hein L, Hannawacker A, Lohse MJ. Novel single chain cAMP sensors for receptor-mediated signal propagation. J Biol Chem. 2004; 279: 37215–37218. 15231839 10.1074/jbc.C400302200

[31] Suzuki Y, Zhang H, Saito N, Kojima I, Urano T, Mogami H. GLP-1 activate protein kinase C though Ca^2+^-dependent activation of phospholipase C in insulin-secretion cells. J Biol Chem. 2006; 281: 28499–28507. 16870611 10.1074/jbc.M604291200

[32] Nagasawa M, Kojima I. Translocation of TRPV2 channel induced by focal administration of mechanical stress. Physiol Rep. 2015; 3: e12296 10.14814/phy2.12296 25677550 PMC4393204

[33] Adachi E, Kazoe Y, Sato Y, Suzuki Y, Urano T, Ueyama T, et al A technique for monitoring multiple signals with a combination of prism-based total internal reflection fluorescence microscopy and epifluorescence microscopy. Pflugers Arch. 2009; 456: 227–234. 10.1007/s00424-009-0705-8 19680684

[34] Hirokawa K, Yamada Y, Matsuda K, Kobayashi M, Hashimoto T, Matsuura M, et al Spontaneous network activity visualized by ultrasensitive Ca^2+^ indicators, yellow Cameleon-nano. Nat Methods. 2010; 7: 729–732. 10.1038/nmeth.1488 20693999

[35] Bleasdale JE, Thakur NR, Gremban RS, Bundy GL, Fitzpatrik FA, Smith RJ, et al Selective inhibition of receptor-coupled phospholipase C-dependent processes in human platelets and polymorphonuclear neutrophils. J Pharmacol Exp Thr. 1990; 255: 756–768. 2147038

[36] Takasaki J, Saito T, Taniguchi M, Kawasaki T, Moritani T. A novel G_q/11_-selective inhibitor. J Biol Chem. 2004; 279: 47438–47445. 15339913 10.1074/jbc.M408846200

[37] Hamano K, Nakagawa Y, Ohtsu Y, Li L, Medina J, Tanaka Y, et al Lactisole: an inhibitor of the glucose-sensing receptor T1R3 expressed in pancreatic β-cells. J Endocrinol. 2015; 226: 57–66. 10.1530/JOE-15-0102 25994004

[38] Hellman B, Grapengiesser E. Glucose-induced inhibition of insulin secretion. Acta Physiol. 2014; 210: 479–488. 10.1111/apha.12217 24354538

[39] Ohtsu Y, Nakagawa Y, Nagasawa M, Takeda S, Arakawa H, Kojima I. Diverse signaling systems activated by the sweet taste receptor in human GLP-1-secreting cells. Mol Cell Endocrinol. 2014; 394: 70–79. 10.1016/j.mce.2014.07.004 25017733

[40] Yaekura K, Yada T. Cytosolic Ca2+-reducing action of cAMP in rat pancreatic β-cells: involvement of thapsigargin-sensitive stores. Am J Physiol. 1998; 274: C513–C521. 9486142 10.1152/ajpcell.1998.274.2.C513

[41] Fridlyand LE, Harbeck MC, Roe MW, Philipson LH. Regulation of cAMP dynamics by Ca^2+^ and G protein-coupled receptors in the pancreatic β-cell: a computational approach. Am J Physiol. 2007; 293: C1924–C1933. 10.1152/ajpcell.00555.2006 17928534

[42] Ganesan S, Calle R, Zawalich K, Greenwalt K, Zawalich W, Shulman GI, et al Immucytochemical localization of alpha-protein C in rat pancreatic bêta-cells during glucose-induced insulin secretion. J Cell Biol. 1992; 119: 313–324. 1400576 10.1083/jcb.119.2.313 PMC2289651

[43] Mogami H, Zhang H, Suzuki Y, Urano T, Saito N, Kojima I, et al Decoding of short-lived Ca^2+^ influx signals into long term substrate phosphorylation through activation of two distinct classes of protein kinase C. J Biol Chem. 2003; 278: 9896–9904. 12514176 10.1074/jbc.M210653200

[44] Wolf BA, Easom RA, McDaniel ML, Turk J. Diacylglycenol synthesis de novo from glucose by pancreatic islets isolated from rats and humans. J Clin Invest. 1990; 85: 482–490. 2405021 10.1172/JCI114463 PMC296449

[45] Geraedts MC, Takahashi T, Vigues S, Markwardt MC, Nkobena A, Cockerham RE, et al Transformation of postingestive glucose responses after deletion of sweet taste receptor subunits or gastric bypass surgery. Am J Physiol. 2012; 303: E464–E474. 10.1152/ajpendo.00163.2012 PMC3423100 22669246

[46] Matschinsky FM. Glucokinase as glucose sensor and metabolic signal generator in pancreatic β-cells and hepatocytes. Diabetes. 1990; 39: 647–652. 2189759 10.2337/diab.39.6.647

[47] Kyriazis GA, Smith KR, Tyberg B, Hummain T, Pratly RE. Sweet taste receptor regulates basal secretion and contribute to compensatory insulin hypersecretion during the development of diabetes in male mice. Endocrinology. 2014; 155: 2112–2121. 10.1210/en.2013-2015 24712876 PMC4020927

[48] Kyriazis GA, Soundarapandian MM, Tyberg B. Sweet taste receptor signaling in β-cells mediates fructose-induced potentiation of glucose-stimulated insulin secretion. Proc Natl Acad Sci USA. 2012; 109: E524–E532. 10.1073/pnas.1115183109 22315413 PMC3286985

[49] Murovets VO, Bachmanov AA, Zolotarev VA. Impaired glucose metabolism in mice lacking Taslr3 taste receptor gene. PLoS ONE. 2015; 10: e130997. 10.1371/journal.pone.0130997 PMC4479554 26107521

